# Resistance exercise training at different loads in frail and healthy older adults: A randomised feasibility trial

**DOI:** 10.1016/j.exger.2021.111496

**Published:** 2021-10-01

**Authors:** Rebecca Marshall-McKenna, Evan Campbell, Frederick Ho, Matthew Banger, Jane Ireland, Philip Rowe, Christine McAlpine, Kate McArthur, Terence J. Quinn, Stuart R. Gray

**Affiliations:** a Institute of Cardiovascular and Medical Sciences, University of Glasgow, Glasgow G12 8TA, United Kingdom of Great Britain and Northern Ireland; b Healthcare Improvement Scotland, Glasgow, United Kingdom of Great Britain and Northern Ireland; c Institute of Health and Wellbeing, University of Glasgow, Glasgow G12 8RZ, United Kingdom of Great Britain and Northern Ireland; d Biomedical Engineering, Graham Hills Building, University of Strathclyde, Glasgow G1 1QE, United Kingdom of Great Britain and Northern Ireland; e Clinical Research Facility, Glasgow Royal Infirmary, Glasgow, United Kingdom of Great Britain and Northern Ireland; f Department of Medicine for the Elderly, Glasgow Royal Infirmary, Glasgow, United Kingdom of Great Britain and Northern Ireland; g Department of Health Promotion and Rehabilitation, Lithuanian Sports University, Kaunas, Lithuania

**Keywords:** Older adults, Frailty, Resistance training, Sarcopenia, Feasibility

## Abstract

**Objectives:**

This trial aimed to determine the feasibility of recruitment, retention, adherence, and safety of a resistance training (RT) intervention to skeletal muscle failure in both frail and non-frail older adults.

**Design:**

An 8-week randomised feasibility trial.

**Setting and participants:**

Older adults, with and without frailty, recruited from both clinics and community.

**Methods:**

Recruitment was based on the number of participants enrolled from those provided with a Patient Information Sheet (PIS). Retention was based on the number of participants who completed the trial. Adherence was based on the number of RT sessions attended out of 16. Outcomes included frailty (Fried criteria), muscle strength (maximal voluntary contraction), functional abilities (Short Physical Performance battery), quality of life (EQ-5D-5L), activities of daily living (LIADL) and safety (diary).

**Results:**

Recruitment target (*n* = 60) was achieved within 15 months, 58 were randomised to high (*n* = 30) or low repetition-load (*n* = 28) groups. Mean age of participants was 72 years (range 65–93). Adherence and retention rate for the RT intervention was ≥70%. There was one serious adverse experience due to the RT intervention. There were no differences (*P* > 0.05) in effects of RT on outcome variables between low and high repetition-load groups.

**Conclusions and implications:**

Recruitment of frail people was challenging. Older adults performing supervised RT to skeletal muscle failure was feasible and safe, with appropriate caution, and the repetition-load did not appear to influence its efficacy. Future research into the effectiveness of this simplified model of RT is warranted.

## Highlights

• Resistance exercise is the most effective way to increase muscle mass.
• This is particularly important in older adults.
• Recruitment of older adults, particularly with frailty, is challenging.
• We found recruitment of older adults feasible, but less so for those with frailty.
• The load for exercise did not appear to influence efficacy of exercise.

## 1 Background

Around the age of 40–50 years, skeletal muscle mass and function decline (Nelson and Fiatarone, 1994), with the loss of skeletal muscle function occurring at a threefold greater rate than skeletal muscle mass (Morat et al., 2016). Impaired skeletal muscle function, in particular low skeletal muscle strength, instead of low skeletal muscle mass, is now considered the principal identifier of sarcopenia in clinical practice (Cruz-Jentoft et al., 2018). Sarcopenia increases the likelihood of falls, decreases functional independence, and reduces quality of life (Beaudart et al., 2017), subsequently, loss of skeletal muscle mass and skeletal muscle strength in sarcopenia has a significant overlap with the physical phenotype of frailty (Fried et al., 2001).

In the UK, the annual excess health care costs associated with skeletal muscle weakness, not sarcopenia per se, has been estimated at £2.5 billion (Pinedo-Villanueva et al., 2019), yet healthcare costs may be higher as most studies have focused on hospitalisation rates (Bruyere et al., 2019). In the UK, the prevalence of sarcopenia is up to 34% in geriatric medicine outpatients (Reijnierse et al., 2015). Older adults in the community with declining muscle strength, a clinically silent process (Rasiah et al., 2020), may result in this cost being higher. The most effective way to increase muscle strength, mass and function is through resistance exercise training (RT), which is effective in older adults, with or without frailty/sarcopenia (Lopez et al., 2018; Vlietstra and Hendrickx, 2018), although adverse events are scarcely reported in studies of older people (Lopez et al., 2018). Current recommendations for RT in older adults (Fragala et al., 2019) suggest performing 1–3 sets, 8–15 repetitions, 2–3 days/week at a load of 70–85% one-repetition maximum (1RM), with modifications for those with frailty. Lower load RT and supervised RT sessions of 1–6 days/week at a lesser load of 30–70% of 1RM (Lopez et al., 2018), may be beneficial for those who are concerned with fear of injury or prefer less challenging loads (Van Roie et al., 2015). However, a simplified model of exercise prescription may be to simply perform RT to volitional muscle failure (VMF) with less focus on load (Mcleod et al., 2019; Al-Ozairi et al., 2021),but which retains the many benefits of resistance exercise. Indeed, data in younger people demonstrated that such exercise is effective regardless of the load used (Burd et al., 2010; Morton et al., 2016; Stefanaki et al., 2019), yet this has not been studied in older adults. Therefore, the aims of this trial were to determine the feasibility of recruitment, retention, adherence, and safety to a RT intervention at high and low repetition-loads to VMF, in frail and non-frail adults.

## 2 Methodology

The trial methods were informed by the guidance set out in the CONSORT extension for randomised pilot and feasibility trials (Eldridge et al., 2016).

### 2.1 Study design

A two-arm, randomised feasibility trial investigated recruitment, retention, adherence, safety of RT, to VMF at different intensities among a population of older adults. Approved by London – Surrey Borders Research Ethics Committee and registered on clinicaltrials.gov (*redacted*).

### 2.2 Recruitment

Inclusion criteria: Aged 65+ years and agree to be randomised. Exclusion criteria: unable to stand, walk and transfer independently from a taxi; already performing RT; unable to provide written consent or deemed unsafe to exercise by their clinician and/or research team.

The intention was to recruit frail patients through the *redacted* Outpatient older people's services and to recruit non-frail adults via newspaper adverts. If a patient was considered suitable/agreeable, a participant information sheet (PIS) was provided, and the trial co-ordinator contacted. The newspaper advert sign posted people to contact the trial co-ordinator for a PIS. The trial co-ordinator contacted those who received a PIS, discussed the study and answered questions. Eligible individuals were invited to the Clinical Research Facility at *redacted* where the trial was conducted, and written consent obtained.

### 2.3 Procedure of assessments

The Research Physiotherapists (EC, RMM) conducted assessments before and post-intervention. Assessments consisted of two appointments; (1) medical history; current medication; falls within last 12 months (participant recall); co-morbidities via the Charlson Comorbidity index (CCI) (Charlson et al., 1987); assessment of frailty status utilised the Fried criteria (Fried et al., 2001); cognitive function using the Montreal Cognitive Assessment (MoCA) (Nasreddine et al., 2005), quality of life questionnaires and muscle strength tests; (2) functional assessments using the Short Physical Performance Battery (SPPB) (Freiberger et al., 2012), and motion analysis to measure the biomechanics of participants activities of daily living such as climbing up and down stairs, and responses to gait perturbances during treadmill walking (data not presented in this manuscript). Participants were asked to wear an accelerometer for seven days and to complete a four-day food diary.

Recruitment, retention and adherence rates to the intervention were calculated as percentages. Recruitment was the number of participants enrolled out of the target sample size. Retention was the number of enrolled participants who completed the trial. Adherence was the number of RT sessions attended out of 16. The pre-determined criteria to proceed with a future trial was 80% recruitment rate within six months of the trial commencing, a 70% retention rate, and 70% adherence rate for both the RT groups. A weekly safety diary was completed with the participant to record general health issues (falls, medication changes or use of healthcare services)that occurred during participation in this study.

### 2.4 Randomisation

After baseline assessment participants were randomised, by a colleague independent of the trial using Graphpad Quickcalcs (https://www.graphpad.com/quickcalcs/randomise1.cfm), stratified by frailty to either high (70% of 1RM) or low (30% of 1RM) repetition-load groups using two sets of pre-prepared opaque sealed envelopes, one set for frail participants and one set for non-frail, this was to ensure similar allocation between the two groups.

### 2.5 Resistance training intervention

The RT intervention was 8 weeks, two individual sessions per week, supervised by a physiotherapist. The intervention targeted lower limb strengthening due to its relevance for maintaining functional activities of daily living (Lopez et al., 2018). Training sessions commenced with a warmup of walking and sit to stand chair exercises, each for 60 s. In the first four sessions participants were familiarised with the equipment (Optima Series, Life Fitness™) and the four exercises; knee extension (KE), leg press (LP), leg curl (LC) and calf press (CP). The physiotherapist discussed safety aspects, good posture, breathing techniques and gradually increased the repetition-load. The LC exercise was removed as participants found this too hard to complete with correct form. The lowest weight was 7 kg on knee extension, ankle weights were initially used for those requiring less repetition-load. By the fifth session participants worked at their allocated repetition-load and were encouraged to work to VMF. Duration of sessions/repetitions varied, and any reports of pain were recorded. The 1RM was reassessed halfway through the intervention and repetition-load adjusted accordingly.

### 2.6 Outcome measures

Skeletal muscle strength assessed via the measurement of 1-RM of the KE, LP and CP and via measurement, with a digital myometer (https://www.mie-uk.com/pgripmyo/), of maximal isometric torque during a maximal voluntary contraction (MVC) of the knee extensor and flexor muscles. Grip strength was measured using the Jamar dynamometer (Beaudart et al., 2016). The Short Physical Performance Battery (SPPB) (Freiberger et al., 2012) was also performed. Ultrasound was used to measure muscle thickness of the vastus lateralis muscle at the midpoint of the thigh (50% of the difference between the trochanterion and tibiale laterale). Physical activity was measured using the activPAL3™ micro (PAL Technologies Ltd., Glasgow, UK) for a 7-day period. Motion analysis was carried out (data to be reported in a subsequent publication).

The following questionnaires were completed: the EQ-5D-5L (Herdman et al., 2011), the Lawton Instrumental Activities of Daily Living (LIADL) (Lawton and Brody, 1969), the Barthel Index of activities of daily living (ADL) (Collin et al., 1988) and the Life Curve™ (Gore et al., 2018). Food intake of each participant was recorded on a four-day diary and analysed using WISP 4.0 dietary analysis software.

Four focus groups (FG) were conducted by members of the research team (EC/RMM) and an external academic colleague (KM), experienced in qualitative research (data to be reported in a subsequent publication).

### 2.7 Sample size

A formal sample size calculation was not performed. To inform the design of a future definitive RCT a recruitment target of 60 participants (30 per arm) was set (Billingham et al., 2013).

### 2.8 Statistical analysis

Rates of recruitment, retention and adherence are presented using descriptive statistics. Baseline differences between the two groups were compared using two-sample *t*-tests (for continuous variables) and fisher's exact test (for categorical variables). In the general linear models, time (pre- and post-intervention), group assignment, and time x group interactions were the independent variables. In addition, frailty x time interactions were also analysed to examine whether frailty may moderate the intervention effectiveness. No correction for multiplicity in data were carried out in this feasibility trial. Missing data was handled using complete data analysis. All analyses were conducted using R version 4.0.2.

## 3 Results

### 3.1 Baseline characteristics

The mean age of participants was 72.2 years (SD 6.3 years) and 63% were female. No differences were seen at baseline between the two groups (Table 1).

**Table 1:** Baseline characteristics.

Characteristics	High load (*N* = 30)	Low load (*N* = 28)	*p*-Value
Age M (SD, min-max)	70.9 years (6.1, 65–92)	73.1 years (5.0, 67–85)	0.15
Sex			0.95
Male	12	10	
Female	18	18	
Body mass index M (SD)	27.9 (5.5)	28.7 (5.0)	0.60
Education M (SD)	13.6 years (2.8)	12.6 years (2.5)	0.16
SIMD status median, mode)	3, 5	2, 1	
MoCA M (SD, min-max)	26.0 (3.8, 14–30)	26.9 (2.4, 21–30)	0.31
CCI M (SD, range)	1.1 (0.2, 4)	0.5 (1.1, 4)	0.09
Falls M (SD, range)	2.6 (6.3, 30)	0.8 (1.5, 6)	0.13
Nutrition (daily)	M (SD)	M (SD)	
Total energy Kcal	1878 (590)	1893 (360)	0.91
Total fat g	72 (32)	76 (15)	0.55
Carbohydrate g	226 (85)	221 (52)	0.82
Sugars g	98 (54)	99 (38)	0.91
Protein g	78 (25)	79 (18)	0.82
Alcohol g	7 (101)	7 (12)	0.99
	M (SD)	M (SD)	
Left quadriceps MVC (Nm)	104 (52)	109 (56)	0.76
Right quadriceps MVC (Nm)	105 (58)	117 (60)	0.51
Left hamstrings MVC (Nm)	44 (19)	48 (24)	0.52
Right hamstrings MVC (Nm)	48 (16)	48 (21)	0.97
Knee extension (Kg) 1RM	40 (27)	47 (20)	0.26
Leg press (Kg) 1RM	58 (25)	60 (17)	0.73
Calf press (Kg) 1RM	54 (19)	49 (16)	0.34
Left VL thickness (mm)	18.5 (3.9)	18.5 (4.0)	0.99
Right VL thickness (mm)	18.5 (3.5)	18.9 (4.5)	0.67
SPPB total score	9.8 (2.9)	10.1 (3.1)	0.76
SPPB 4 m gait time	4.6 (2.7)	3.9 (1.5)	0.23
SPPB 5× STS time	11.3 (4.1)	10.8 (2.9)	0.69
EQ-5D-5L index	1.4 (3.3)	0.82 (0.2)	0.35
EQ-5D-5L VAS	78.6 (20.9)	80.8 (14.9)	0.61
Barthel index	19.63 (1.16)	19.71 (0.7)	0.34
LIADL	7.5 (1.5)	7.7 (0.9)	0.47
LifeCurve™	2.04 (3.7)	0.78 (2.7)	0.16
ActivPAL3 daily steps	9135 (5337)	8265 (3980)	0.50

N (Number of participants), M (Mean), SD (Standard Deviation), SIMD (Scottish Index of Multiple Deprivation), MoCA (Montreal Cognitive Assessment), CCI (Charlson Comorbidity Index), Falls (over the past 12 months), MVC (Maximum voluntary contraction), (n) newtons, 1RM (one repetition maximum), VL (vastus lateralis).

### 3.2 Total exercise load

The average total exercise load (repetitions*load) during each session was for the knee extensor exercise 441(270) kg in the high repetition load and was 509(370) kg in the low repetition group (*p* = 0.48). For the leg press the average total exercise load in each session was 1837(1082) kg in the high repetition load and was 6316(3681) kg in the low repetition group (*p* < 0.001). For the calf press the average total exercise load in each session was 1582(587) kg in the high repetition load and was 5527(3539) kg in the low repetition group (p < 0.001).

### 3.3 Recruitment, retention and adherence

The trial ran between 13.08.18 and 19.12.19. The first participant was recruited on 21.08.18, and 80% (*n* = 48) recruitment was achieved on 25.06.19 (10 months). The trial had a retention rate of 75% (45/60) and an adherence rate of 75.8% (364/480) (high repetition-load group) and 78.4% (351/448) (low repetition-load group). Thus, two of the three pre-determined criteria were achieved.

**Fig. 1:**
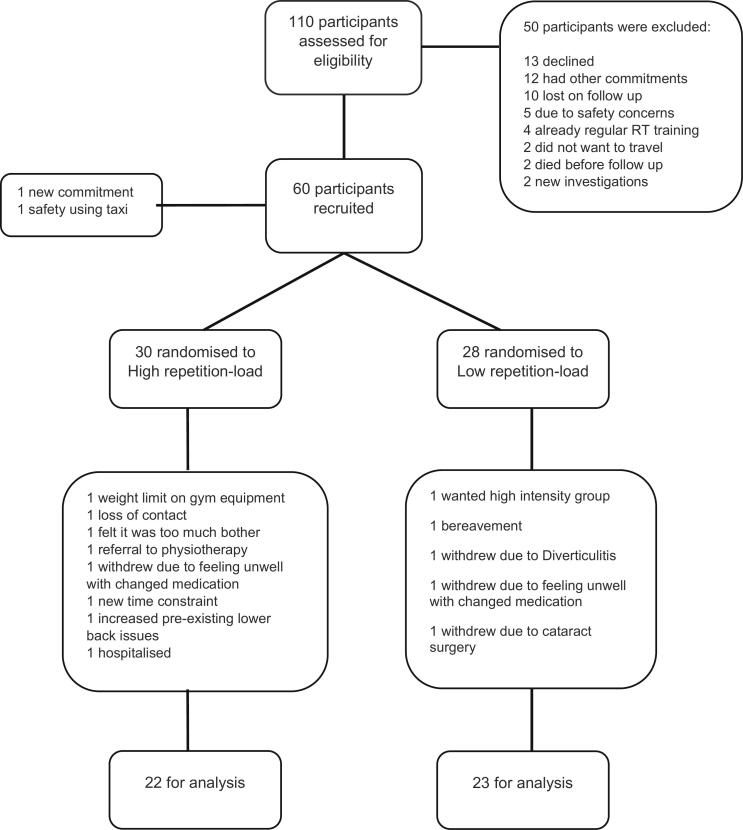
CONSORT flow diagram displaying movement of participants.

Patients (*n* = 25) were approached and provided with a PIS at outpatient clinics, of which, 40% (*n* = 10) were recruited (6 frail, 2 pre-frail, 1 non-frail). Members of the public (*n* = 85) contacted the trial co-ordinator following the newspaper adverts, of which 50 (59%) were recruited (31 non-frail,18 pre-frail). Two participants withdrew before randomisation (unable to transfer safely from a taxi; unforeseen commitments) (Fig. 1). Thus, 58 participants (6 frail, 20 pre-frail, 32 non-frail) were randomised to high (*n* = 30) and low repetition-load groups (*n* = 28). During RT training a total of 13 participants withdrew (4 frail, 4 pre-frail, 5 non-frail) (Fig. 1).

### 3.4 Safety diary

A serious adverse event (due to hospitalisation) was reported by one participant who had pneumonia during the Christmas holiday period, and was not related to the intervention. Overall, 22 participants had at least one entry recorded in the safety diary; 15 appointments for blood tests with GP/Nurse, medication changes (*n* = 4), chest infections requiring antibiotics (*n* = 3), follow up appointments with consultants (n = 4). During the intervention period, six participants reported falls, two at home and four on ice, all completed the trial.

In relation to the intervention,– one participant became hypotensive during post-assessment and was reviewed by staff and hospitalised for observation of bradycardia. Participants from both groups experienced several adverse experiences, of which three were expected experiences of RT; mechanical low back pain-*low load*; knee pain *high* *load*; delayed onset of muscle soreness-*low load*; and four unexpected experiences of RT; inflammation of talocrural joint, lateral hip pain, lower leg oedema-*low load*; and skin hypersensitivity over lower tibia-*high load*.

A total of 17 participants reported pain (*n* = 9 high load, *n* = 8 low load), of which, the KE exercise was the most frequent cause. Fifteen of these participants successfully completed the trial and two were withdrawn by the physiotherapist (change in medication causing fatigue and exacerbation of pre-existing back issues).

### 3.5 Outcome measures

Of the 58 participants recruited, data for 45 participants were available for analysis (Fig. 1). Missing data was due to ill health (participant/researcher) or withdrawing from the intervention. The food diary was the most frequent incomplete outcome measure (*n* = 10).

Following the RT intervention outcome measures were repeated, and no significant differences were observed between the raw scores of the two groups (Table 2).

**Table 2:** Post intervention outcome measures.

Post-Intervention	High Load (*n* = 22)	Low Load (*n* = 23)	p
	Mean (SD)	Mean (SD)	
Body mass index	27.0 (4.1)	28.7 (4.9)	0.22
Frailty score	0.27 (0.55)	0.50 (0.80)	0.28
Left quadriceps MVC (Nm)	119 (47)	128 (55)	0.54
Right quadriceps MVC (Nm)	116 (53)	134 (60)	0.29
Left hamstrings MVC (Nm)	47 (17)	47 (24)	0.94
Right hamstrings MVC (Nm)	53 (16)	52 (24)	0.90
Knee extension (Kg) 1RM	56 (29)	53 (21)	0.68
Leg press (Kg) 1RM	69 (25)	66 (22)	0.68
Calf press (Kg) 1RM	66 (22)	60 (16)	0.32
Left VL thickness (mm)	19.9 (3.8)	19.0 (3.8)	0.48
Right VL thickness (mm)	19.9 (3.7)	20.1 (3.6)	0.88
SPPB total score	11.1 (2.0)	10.6 (2.2)	0.39
SPPB 4 m gait time	3.5 (1.0)	3.6 (1.0)	0.68
SPPB 5xSTS time	9.5 (3.3)	13.3 (10.4)	0.11
Right grip (Kg)	27.4 (8.8)	26.9 (11.3)	0.87
Left grip (Kg)	27.0 (10.0)	26.7 (11.2)	0.93
EQ5D5L Index	0.9 (0.1)	38.9 (178.3)	0.34
EQ5D5L VAS	88.6 (9.3)	82.8 (14.3)	0.12
Barthel Index	20.0 (0.9)	19.8 (0.5)	0.10
LIADL	7.9 (0.3)	8.0 (0.2)	0.53
LifeCurve™	0.68 (1.60)	0.73 (1.75)	0.94
ActivPAL 3 daily steps	8905 (5089)	9198 (5651)	0.87

BMI (Body Mass Index), MVC (Maximum voluntary contraction), (n) newtons, 1RM (one repetition maximum), VL (vastus lateralis), STS (sit to stand).

### 3.6 Effects of exercise load on adaptations to resistance exercise training

Data were explored by load by fitting a load-time interaction effect to the model (Table 3). There were no group or interaction effects found for any outcome measures. There was a significant time effect between baseline and post-intervention for: muscle strength measurements of KE 1RM (*p* < 0.02); CP 1RM (*p* < 0.03) and 4 m walking time (p < 0.03). Similarly, time effects were found for the frailty score (*p* < 0.04) and the QoL EQ-5D-5L VAS (p < 0.03).

**Table 3:** Effects of RT load on time and group-time interaction.

Measure	Time β-coefficient (95% CI)	P	Group β-coefficient (95% CI)	P	Group x time β-coefficient (95% CI)	P
BMI	-0.90 (−3.62, 1.81)	0.52	0.73 (−1.84, 3.30)	0.58	0.97 (−2.94, 4.88)	0.63
Frailty score	-0.59 (−1.17, −0.02)	0.04*	0.03 (−0.51,0.56)	0.92	0.20 (−0.61, 1.02)	0.63
Left Quadriceps MVC (Nm)	14.77 (−15.99, 45.53)	0.35	4.91 (−25.85, 35.67)	0.76	4.56 (−39.18, 48.30)	0.84
Right Quadriceps MVC (Nm)	10.77 (−22.97, 44.50)	0.53	11.53 (−22.21, 45.27)	0.50	6.90 (−41.07, 54.88)	0.78
Left Hamstrings MVC (Nm)	3.14 (−8.48, 14.77)	0.60	3.64 (−7.25, 14.52)	0.51	-4.12 (20.68, 12.45)	0.63
Right Hamstrings MVC (Nm)	5.24 (−5.50, 15.99)	0.34	0.22 (−9.84, 10.27)	0.97	-1.01 (−16.32, 14.30)	0.90
Knee Extension (Kg) 1RM	15.78 (2.63, 28.92)	0.02*	6.33 (−5.14, 17.81)	0.28	-10.41 (−29.15, 8.33)	0.28
Leg press (Kg) 1RM	11.73 (−0.69, 24.15)	0.07	1.97 (−8.75, 12.69)	0.72	-5.05 (−22.58, 12.49)	0.57
Calf press (Kg) 1RM	12.03 (1.53, 22.53)	0.03*	−5.23 (−14.34, 3.88)	0.26	-1.68 (−16.67, 13.30)	0.83
Left VL thickness (mm)	1.37 (−0.82, 3.57)	0.22	0.01 (−1.99, 2.01)	0.99	-0.86 (−3.96, 2.25)	0.59
Right VL thickness (mm)	1.47 (−0.73, 3.66)	0.19	0.46 (−1.54, 2.45)	0.66	-0.28 (−3.38, 2.82)	0.86
SPPB total score	1.31 (−0.17, 2.79)	0.09	0.24 (−1.13, 1.61)	0.73	-0.79 (−2.89, 1.31)	0.46
SPPB 4 m gait time	-1.11 (−2.10, −0.13)	0.03*	−0.69 (−1.60, 0.22)	0.14	0.82 (−0.58, 2.21)	0.26
SPPB 5× STS time	-1.78 (−5.14, 1.58)	0.30	−0.41 (−3.65, 2.83)	0.80	4.26 (−0.53, 9.05)	0.08
Right grip (Kg)	3.06 (−2.46, 8.58)	0.28	0.65 (−4.51, 5.82)	0.80	-1.17 (−9.04, 6.69)	0.77
Left grip (Kg)	3.24 (−2.84, 9.32)	0.30	1.97 (−3.73, 7.66)	0.50	-2.27 (−10.93, 6.40)	0.61
EQ5D5L Index	-0.49 (−46.76, 45.78)	0.98	−0.59 (−43.32,42.14)	0.98	38.53 (−26.95, 104.00)	0.25
EQ5D5L VAS	10.22 (1.31, 19.13)	0.03*	2.44 (−5.79, 10.66)	0.56	-8.22 (−20.75, 4.30)	0.20
Barthel Index	0.37 (−0.06, 0.80)	0.010	0.08 (−0.32, 0.48)	0.69	-0.26 (−0.87, 0.35)	0.40
LIADL	0.42 (−0.10, 0.94)	0.11	0.23 (−0.25, 0.71)	0.35	-0.18 (−0.91, 0.55)	0.63
LifeCurve™	-1.35 (−2.92, 0.22)	0.09	−1.26 (−2.68, 0.16)	0.09	1.30 (−0.88, 3.48)	0.25
ActivPAL3 daily steps	−229.65 (−3226.76, 2767.46)	0.88	−870.19 (−3555.96, 1815.58)	0.53	1162.46 (−3026.80, 5351.71)	0.59

BMI (Body Mass Index), MVC (Maximum voluntary contraction), (n) newtons, 1RM (one repetition maximum), VL (vastus lateralis), STS (sit to stand).

**Table 4:** Effects of frailty status on frailty-time interaction.

Outcome	Time β-coefficient (95% CI)	P	Frailty Status β-coefficient (95% CI)	P	Time x Frailty Status β-coefficient (95% CI)	P
BMI	−1.08 (−3.60, 1.44)	0.40	1.07 (−1.47, 3.62)	0.41	1.79 (−2.13, 5.71)	0.37
Frail Score	0.08 (−0.27, 0.43)	0.67	1.96 (1.61, 2.31)	0.0001***	−1.21 (−1.74, −0.67)	0.0001***
Left Quadricep MVC (Nm)	15.46 (−12.86,43.78)	0.29	−18.20 (−49.02, 12.62)	0.25	3.44 (−40.49, 7.38)	0.88
Right Quadricep MVC (Nm)	7.68 (−23.76,39.11)	0.63	−19.10 (−53.31, 15.12)	0.28	15.69 (−33.07,64.46)	0.53
Left Hamstring MVC (Nm)	1.97 (−8.76,12.69)	0.72	−7.34 (−18.07, 3.38)	0.18	−2.71 (−19.15, 13.73)	0.75
Right Hamstring MVC (Nm)	6.07 (−3.93, 16.07)	0.24	−3.92 (−13.92, 6.07)	0.44	−3.61 (−18.93, 11.71)	0.64
Knee Extension (Kg)1RM	8.31 (−3.98, 20.60)	0.19	−9.47 (−20.93, 1.99)	0.11	4.84 (−13.95, 23.64)	0.61
Leg Press (Kg) 1RM	5.84 (−5.58, 17.26)	0.32	−12.10 (−22.64, −1.56)	0.03*	6.83 (−10.46, 24.12)	0.44
Calf Press (Kg) 1RM	9.29 (−0.49, 19.06)	0.06	−9.80 (−18.92, −0.68)	0.04*	4.15 (−10.84, 19.14)	0.59
Left VL thickness (mm)	0.33 (−1.70, 2.35)	0.75	−0.96 (−2.97, 1.04)	0.35	1.42 (−1.73, 4.57)	0.38
Right VL thickness mm)	0.55 (−1.46, 2.56)	0.59	−0.37 (−2.36, 1.62)	0.72	1.96 (−1.16, 5.09)	0.22
SPPB total score	0.44 (−0.82, 1.70)	0.50	−2.49 (−3.74, −1.23)	0.0002***	0.87 (−1.08, 2.82)	0.38
SPPB 4 m gait time	−0.22 (−1.03, 0.59)	0.59	2.07 (1.25, 2.88)	0.0001***	−0.98 (−2.23, 0.28)	0.13
SPPB 5× STS time	−0.42 (−3.45, 2.61)	0.79	2.22 (−1.05, 5.49)	0.19	2.02 (−2.81, 6.85)	0.41
Right grip	1.34 (−3.76, 6.44)	0.61	−4.58 (−9.68, 0.52)	0.08	2.35 (−5.47, 10.17)	0.56
Left grip	0.53 (−5.09, 6.15)	0.85	−5.51 (−11.13, 0.11)	0.06	3.42 (−5.19, 12.03)	0.44
EQ-5D-5L index	32.18 (−10.90,75.27)	0.15	0.47 (−42.62, 43.55)	0.98	−32.76 (−99.45, 33.93)	0.34
EQ-5D-5L VAS	1.05 (−5.75, 7.85)	0.76	−21.52 (−28.39, −14.65)	0.0001***	9.41 (−1.18, 20.01)	0.08
Barthel	0.02 (−118.77,118.82)	1.00	88.10 (−30.69, 206.90)	0.15	−88.24 (−272.10,95.63)	0.35
LIADL	0.00 (−0.44, 0.44)	1.00	−0.92 (−1.37, −0.47)	0.0001***	0.74 (0.05, 1.43)	0.04*
LifeCurve™	−0.25 (−1.58, 1.08)	0.71	2.58 (1.26, 3.91)	0.0002***	−0.98 (−3.02, 1.06)	0.35
ActivPAL3 daily steps	410.19 (−1862.23, 2682.61)	0.72	−4794.57(−7094.27, −2494.57)	0.0001***	-0.98 (−2.23, 0.28)	0.13

BMI (Body Mass Index), MVC (Maximum voluntary contraction), (n) newtons, 1RM (one repetition maximum), VL (vastus lateralis), STS (sit to stand).

The data were also explored by frailty status, regardless of load, by fitting a frailty-time interaction effect to the model (Table A1 and Table 4). No time or frailty-time interaction effects were noted in strength and functional measures. However, there were group differences for muscle strength measurements; calf press 1RM (*p* < 0.04); leg press 1RM (p < 0.03); and functional measures SPPB total score (*p* < 0.0002); 4 m walking time (*p* < 0.0001) and number of steps (p < 0.0001). Group effects were observed for the frailty score (p < 0.0001), EQ-5D-5L VAS (p < 0.0001), LIADL (p < 0.0001) and Life Curve™ (p < 0.0002). Furthermore, there was a frailty-time interaction effect for the frailty score (p < 0.0001) and the LIADL (p < 0.04) with greater increases in those without frailty.

## 4 Discussion

This is the first, to our knowledge, randomised controlled feasibility trial comparing high and low repetition-load RT to VMF, in non-frail and frail older adults. The recruitment target was successfully achieved, albeit slower than anticipated and fewer patients were referred from the outpatient setting. The feasibility of the intervention in terms of adherence and retention, in both groups, were high and met the predefined criteria. Only one participant withdrew due to dissatisfaction following randomisation to the low load arm.

Moreover, RT of the lower limb muscles to VMF was safe, with appropriate caution to age-related changes such as osteoarthritis or thinning skin. Our study reported one serious adverse event due to the intervention, the participant became hypotensive during post-assessment. This could possibly be prevented by advising participants about adequate hydration and nutrition prior to sessions and monitoring the blood pressure of participants pre/post sessions. Our training log recorded 17 participants who stopped exercise due to pain. Yet, in previous literature only one of 16 studies had reported pain (2 subjects) and almost 50% provided no information on any adverse events (Lopez et al., 2018). Therefore, our trial filled an important gap in knowledge around events to consider and will improve awareness when developing future RT interventions with older adults.

As expected, based on data from young participants, there were no apparent differences in the effects of RT, to VMF, on outcomes, when comparing high and low loads. This trial provides some early support to the simplified model of resistance exercise prescription previously suggested (Mcleod et al., 2019) by showing that RT to VMF is feasible and safe, and effects appear similar regardless of the load. Therefore, RT could be prescribed with older adults performing exercise at a load they are comfortable with and without needing to achieve a set number of repetitions, but just to focus on reaching VMF.

This study is not without limitations. Primarily, we did not recruit as many frail individuals to the study. Perceived barriers and acceptability of this intervention was explored in our qualitative work. Some of the issues faced in clinics may be explained by competing clinical priorities, staff turn-over, or lack of research support at clinics. However, 18 people with pre-frailty were recruited via the newspaper adverts suggesting a need for effective interventions to modify and improve this clinically silent process that predisposes older adults to frailty (Rasiah et al., 2020). The outcome measures used were feasible, except for the paper food diary which had a relatively low completion rate. Future work should consider alternative options for collecting nutritional information.

When our data was explored by frailty status, we observed differences in skeletal muscle strength and functional outcomes between the non-frail and frail groups. There were improvements in frailty status and LIADL, thus supporting the potential effectiveness of RT to VMF in frail adults. However, this trial was not powered to detect changes in any of these outcomes, thus further work is needed to compare effectiveness of high and low load RT on clinically relevant outcomes.

## 5 Conclusion

This trial has provided preliminary evidence that performing supervised RT, to VMF, in older adults is feasible, regardless of the repetitive-load, in terms of adherence, retention, and safety. RT performed at different loads did not appear to influence its efficacy, although we were not fully powered to detect such differences, our sample size had 80% power to detect effects of 0.37SD. Improved recruitment strategies of frail patients (e.g., public engagement / research support at clinics) must be considered for future research warranted into the effectiveness of such RT.

The following is the supplementary data related to this article.

Table A1 Baseline and post-intervention outcome measures by frailty status and repetition load Table A1

## CRediT authorship contribution statement

**Rebecca Marshall-McKenna:** Project administration, Writing – Original Draft, Investigation. **Evan Campbell:** Project administration, Writing – Review and Editing, Investigation. **Frederick Ho:** Formal analysis, Writing – Review and Editing. **Matthew Banger:** Writing – Review and Editing, Investigation. **Jane Ireland:** Writing – Review and Editing, Investigation. **Philip Rowe:** Resources, Conceptualization, Funding acquisition, Writing – Review and Editing. **Christine McAlpine**: Writing – Review and Editing, Investigation. **Kate McArthur**: Writing – Review and Editing, Investigation. **Terence J Quinn:** Supervision, Conceptualization, Funding acquisition, Writing – Review and Editing. **Stuart R Gray:** Supervision, Conceptualization, Funding acquisition, Writing – Review and Editing.

## Declaration of competing interest

None to declare.
