# Supplementary material for: Resistance exercise training at different loads in frail and healthy older adults: A randomised feasibility trial
Source: Exp Gerontol. 2021 Oct 1;153:111496. doi: 10.1016/j.exger.2021.111496 (PMC8434423; doi:10.1016/j.exger.2021.111496)
Supplement: Table A1 — Baseline and post-intervention outcome measures by frailty status and repetition load [file mmc1.docx]

**Table A1 Baseline and post-intervention outcome measures by frailty status and repetition load**

|  | Baseline |  |  |  | Post Intervention |  |  |  |
| --- | --- | --- | --- | --- | --- | --- | --- | --- |
|  | Non-Frail |  | Frail |  | Non-Frail |  | Frail |  |
|  | High Load (n=15) | Low Load (n=12) | High Load (n=7) | Low Load (n=11) | High Load (n=15) | Low Load (n=12) | High Load (n=7) | Low Load (n=11) |
|  | Mean (SD) | Mean (SD) | Mean (SD) | Mean (SD) | Mean (SD) | Mean (SD) | Mean (SD) | Mean (SD) |
| Body Mass Index | 28.1 (6.5) | 27.4 (4.3) | 27.6 (3.5) | 30.0 (5.5) | 26.7 (4.4) | 26.8 (2.6) | 27.7 (3.6) | 30.9 (5.9) |
| Frailty score | 0.00 (0.00) | 0.00 (0.00) | 2.2 (1.3) | 1.8 (1.0) | 0.1 (0.3) | 0.1 (0.3) | 0.7 (0.8) | 0.9 (0.9) |
| Left Quadriceps MVC (Nm) | 110 (42) | 120 (65) | 93 (68) | 98 (47) | 119 (41) | 144 (58) | 119 (62) | 113 (48) |
| Right Quadriceps MVC (Nm) | 110 (47) | 130 (69) | 95 (76) | 103 (50) | 112 (44) | 145 (70) | 124 (72) | 123 (48) |
| Left Hamstrings MVC (Nm) | 49 (17) | 49 (16) | 37 (20) | 46 (31) | 49 (15) | 54 (19) | 43 (21) | 40 (27) |
| Right Hamstrings MVC (Nm) | 49 (14) | 50 (16) | 46 (19) | 45 (26) | 53 (16) | 59 (25) | 53 (19) | 45 (23) |
| Knee Extension (Kg) 1RM | 46 (23) | 51 (19) | 32 (30) | 44 (20) | 56 (26) | 56 (21) | 55 (36) | 49 (21) |
| Leg press (Kg) 1RM | 66 (27) | 63 (18) | 47 (15) | 56 (16) | 73 (29) | 67 (20) | 62 (16) | 66 (24) |
| Calf press (Kg) 1RM | 58 (19) | 55 (16) | 48 (18) | 43 (15) | 67 (24) | 63 (14) | 62 (20) | 56 (17) |
| Left VL thickness (mm) | 18.8 (3.9) | 19.1 (4.0) | 18.0 (3.9) | 17.9 (4.0) | 19.3 (4.3) | 19.2 (4.5) | 21.1 (2.0) | 18.9 (3.2) |
| Right VL thickness (mm) | 18.8 (3.3) | 18.8 (3.0) | 17.9 (4.0) | 19.0 (5.7) | 19.3 (3.9) | 19.5 (3.3) | 21.4 (3.0) | 20.7 (4.0) |
| SPPB total score | 10.9 (2.1) | 11.2 (1.2) | 8.2 (3.2) | 8.9 (4.0) | 11.6 (1.3) | 11.4 (0.8) | 10.0 (3.0) | 9.8 (2.8) |
| SPPB 4m gait time | 3.5 (1.0) | 3.1 (0.3) | 6.2 (3.5) | 4.7 (1.8) | 3.2 (0.6) | 3.1 (0.3) | 4.3 (1.4) | 4.2 (1.1) |
| SPPB 5xSTS time | 9.7 (2.7) | 10.8 (2.5) | 13.9 (4.9) | 10.9 (3.5) | 8.9 (2.7) | 11.0 (2.2) | 10.9 (4.4) | 15.7 (14.5) |
| Right grip (Kg) | 27.1 (7.4) | 26.3 (8.7) | 20.3 (7.6) | 23.8 (14.3) | 27.9 (9.0) | 28.3 (8.9) | 26.4 (8.8) | 25.5 (13.7) |
| Left grip (Kg) | 27.2 (8.3) | 27.2 (9.3) | 18.6 (12.6) | 24.4 (14.2) | 27.2 (9.0) | 28.5 (9.2) | 26.8 (12.6) | 24.9 (13.3) |
| EQ5D5L Index | 0.9 (0.1) | 0.9 (0.1) | 2.2 (5.3) | 0.7 (0.2) | 1.0 (0.1) | 76.9 (252.1) | 0.8 (0.1) | 0.8 (0.2) |
| EQ5D5L VAS | 88.6 (7.4) | 89.9 (9.5) | 63 (25.3) | 71.7 (13.9) | 90.0 (7.9) | 90.5 (8.4) | 85.0 (12.4) | 74.4 (15.0) |
| Barthel Index | 19.9 (0.2) | 19.9 (0.3) | 19.2 (1.8) | 19.5 (0.9) | 20.0 (0.0) | 19.9 (0.3) | 20.0 (0.0) | 19.7 (0.7) |
| LIADL | 8.0 (0.0) | 8.0 (0.00) | 6.6 (2.1) | 7.4 (1.2) | 8.0 (0.0) | 8.0 (0.0) | 7.7 (0.5) | 7.9 (0.3) |
| LifeCurve™ | 0.53 (1.94) | 0.00 (0.00) | 4.36 (4.6) | 1.62 (3.71) | 0.08 (0.28) | 0.00 (0.00) | 2.00 (2.45) | 1.45 (2.30) |
| ActivPAL 3 daily steps | 11055 (5462) | 10403 (3902) | 5509 (2592) | 6279 (2967) | 10176 (4817) | 12591 (5837) | 4457 (3501) | 5427 (1763) |
